# Disappearance of Anterior Cervical Corpectomy Cage

**DOI:** 10.7759/cureus.3985

**Published:** 2019-01-30

**Authors:** Mei-Yin Yeh, Wen-Cheng Huang, Chao-Hung Kuo, Peng-Yuan Chang, Henrich Cheng

**Affiliations:** 1 Neurosurgery, Taipei Veterans General Hospital, Taipei, TWN; 2 Neurosurgery, Tao-Yuan General Hospital, Tao-Yuan, TWN

**Keywords:** anterior cervical fusion, esophageal perforation, cage, dislodgement

## Abstract

Although cage subsidence and dislodgement are not uncommon in anterior cervical spine surgery, missing cages have seldom been reported. This is the first report of the disappearance of a metallic corpectomy cage after anterior cervical fusion.

A 63-year-old man, who had a history of ankylosing spondylitis and diabetes mellitus, was involved in a motor vehicle accident that broke his neck. The traumatic C6 burst fracture caused myelopathy and instability, which required surgery. He then underwent anterior C6 corpectomy with circumferential fixation, including anterior plating and posterior lateral mass screws from C5-C7. There was a significant improvement in neurological function after the surgery and he could ambulate independently. However, upon a visit at six months postoperation, there was dislodgement of the anterior cervical plate and cage. An attempt to revise the anterior fusion construct was made subsequently, but this surgery could only remove the plate. The metallic cage was left in place during the revision surgery because it was firmly incorporated into the C5 and C7 vertebra and could hardly be adjusted intraoperatively. There were no other interventions during the interval. Upon his visit at 23 months after the initial surgery, the metallic cage was missing. No examinations could locate the cage anywhere in the body, including 36-inch radiographs that demonstrated completely the disappearance of the metallic corpectomy cage. The posterior arthrodesis seemed stable and the patient had no dysphagia or any other gastrointestinal symptoms. The process of the disappearance of the corpectomy cage was never noticed by the patient and he remains free of symptoms to date.

The complete dislodgement of a cervical corpectomy cage that was placed anteriorly could happen without symptoms. The cage might have been expelled during bowel movements and caused little problem. Failure to achieve arthrodesis in anterior cervical fusion, therefore, must be closely monitored.

## Introduction

Anterior cervical discectomy and fusion (ACDF) and anterior cervical corpectomy and fusion (ACCF) are both common options for patients with herniated intervertebral disc, spondylosis, trauma, and ossification of the posterior longitudinal ligament (OPLL) [1-3]. Although both ACDF and ACCF have frequently yielded high fusion rates and extraordinarily good patient satisfaction, there nevertheless have been reports of the dislodgement of instrumentation [4-6] or pseudarthrosis occasionally [7-8]. Many of these implant-related complications, such as dysphagia or hoarseness, could cause minor symptoms and be amended during revision surgery [5-6]. However, some could cause serious problems, including esophageal perforation or mediastinitis [1,9]. Due to their relatively low incidence rates, the actual risk factors, adverse effects, and strategies of management have remained elusive. Moreover, the material of the cages might also affect outcomes. The most commonly used materials to replace the vertebral body after corpectomy have been autografts, allografts, and polyether ether ketone (PEEK) cages with bone grafts or extenders [2,10-14]. Although they usually yield high rates of bone fusion, each of the material’s features vary, and thus might differ in the methods of evaluation, management, and prognosis.

This is the first report in the literature on a missing metallic corpectomy cage after ACCF. Serial radiological evaluations, including computed tomography (CT) and magnetic resonance imaging (MRI), were used to demonstrate the process. Fortunately, the disappearance of the cage caused few symptoms and adverse consequences so far. Although there was no evidence of perforation of the oropharyngeal mucosa, it was particularly evident that the corpectomy cage was somehow expelled through the gastrointestinal tract because the metallic cage should be easily found by roentgenological evaluations if it were still inside the body.

## Case presentation

A 63-year-old man, who had had ankylosing spondylitis, was involved in a motor vehicle accident that caused a C6 burst fracture and incomplete spinal cord injury. He presented with marked left paraparesis, particularly weak in his left shoulder and arm. Also, he had had Type 2 diabetes and was wearing a neck collar for cervical spondylosis when the accident took place.

**Figure 1:**
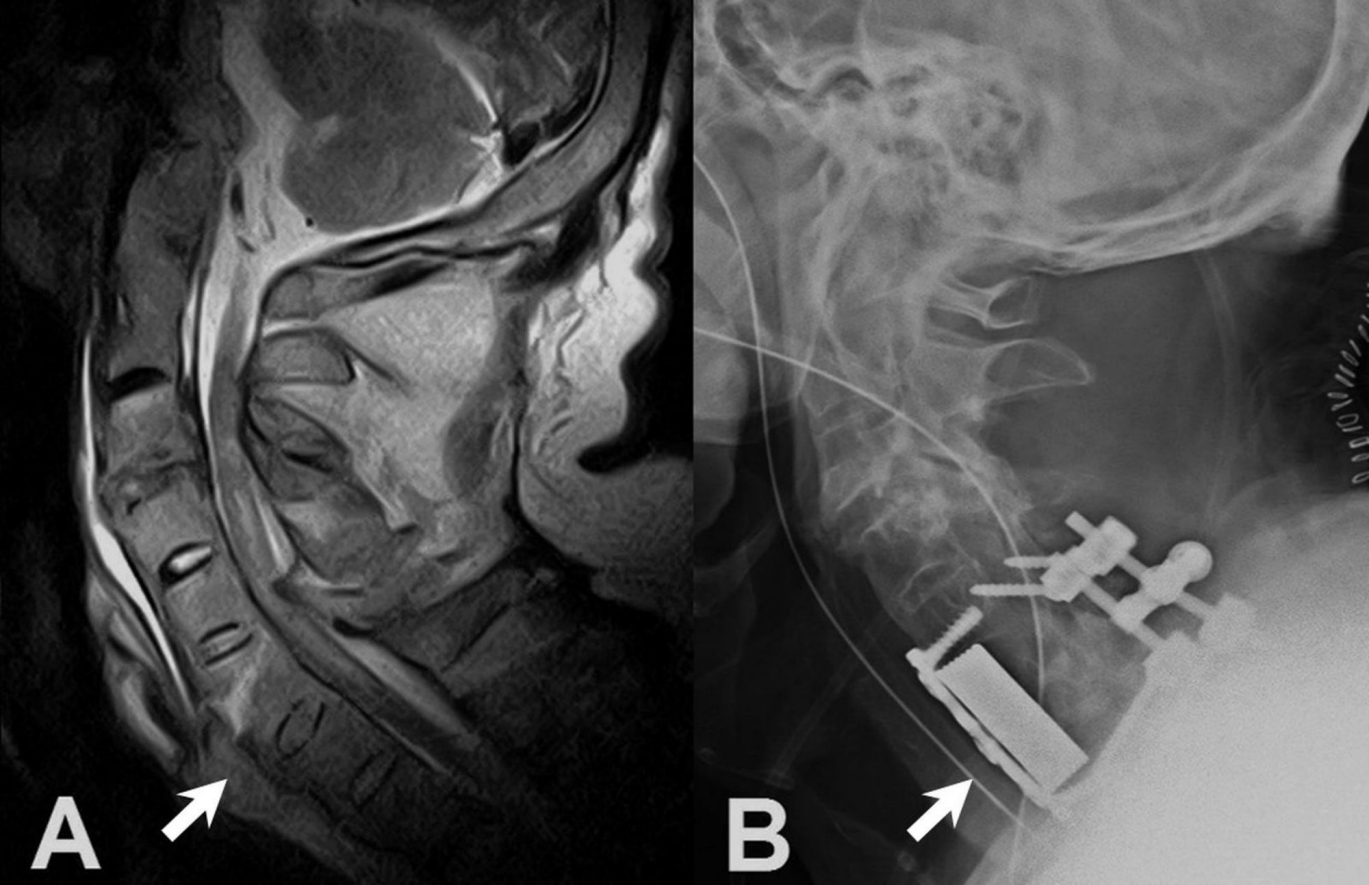
Lateral radiograph: preoperative and postoperative A: Preoperative sagittal T2-weighted cervical magnetic resonance image demonstrated remarkable spinal cord compression with increased intramedullary signal intensity, caused by a C6 burst fracture (arrow), in the subaxial cervical spine. B: Postoperative lateral radiograph demonstrated the instrumented anterior and posterior fusion, C5-6-7.

There was a burst fracture of the C6 and a fracture of the lamina in addition to a herniated disc at C5-6 level, which caused spinal cord compression and increased intramedullary signal intensity on T2 weighted magnetic resonance imaging (MRI) (Figure 1A). In order to improve the spinal alignment, the patient was admitted to the intensive care unit for halo traction prior to surgery. The patient then underwent circumferential decompression and fixation, which involved a corpectomy of C6 and total laminectomies of C3 to C7 with anterior instrumentation of plate and screws and posterior lateral mass screws at C5 and C7 (Figure 1B). The surgery went smoothly and the hospitalization was uneventful. There was a significant improvement in muscle power, and he was able to ambulate at discharge. The patient had been kept in a neck collar postoperation.

**Figure 2:**
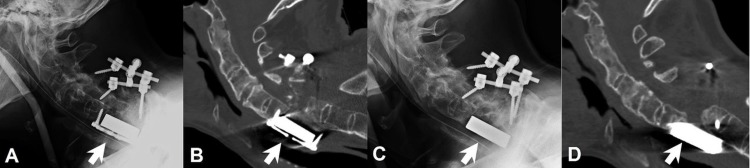
Dislodgement of the plate and cage A: Lateral radiograph taken after the initial surgery. B: Cervical computed tomography (CT) scan at three weeks postoperation, demonstrated the dislodgement of the plate and cage in the prevertebral space. C: Lateral radiograph taken at two months after the second operation, which could only remove the dislodged screws and plate and left the dislocated metallic cage. D: Cervical CT scan taken at four months after the second operation demonstrated that the dislodged cage seemed stabilized after the reoperation.

Three weeks postoperation, there was a sudden onset of right hand weakness, the opposite side of his initial presentation. The CT scan demonstrated plate and cage dislodgement that was anteriorly dislocated over the C7 vertebral body (Figures 2A-2B). There was no dysphagia so we decided to treat him conservatively. He was then put into a halo-vest for immobilization, and his muscle power gradually improved to that of postoperation in a few days. Thus, the patient was discharged to a rehabilitation facility with a halo-vest. At three months postoperation, the patient opted to undergo revision surgery due to the discomfort caused by the halo-vest. Unfortunately, we could only remove the cervical plate rather than restore the corpectomy cage because it was incorporated into the neighboring vertebral bodies, C5 and C7. Under intraoperative inspection, the porous metallic cage was integrated by bony growth and the interfaces between the cage and vertebral bodies were firm and covered by new bone. The lateral radiograph obtained two months after the second operation demonstrated the persistently dislocated metallic cage without any movement (Figure 2C). A CT scan also confirmed that the metallic cage was maintained in an unwanted position four months after the second operation (Figure 2D). The cage seemed to have no movement during the interval period in which the patient was kept in a neck collar and free of symptoms.

**Figure 3:**
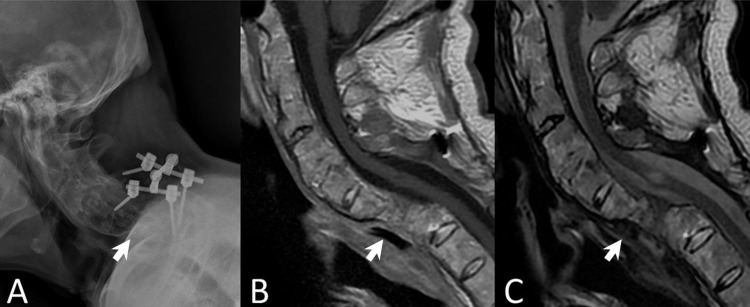
Disappearance of the cage A: Lateral radiograph taken at 22 months after the initial operation. The metallic cage was no longer appreciated. B: Sagittal T1-weighted magnetic resonance imaging (MRI) demonstrated no spinal cord compression. C: Sagittal T2-weighted MRI demonstrated no spinal cord compression.

**Figure 4:**
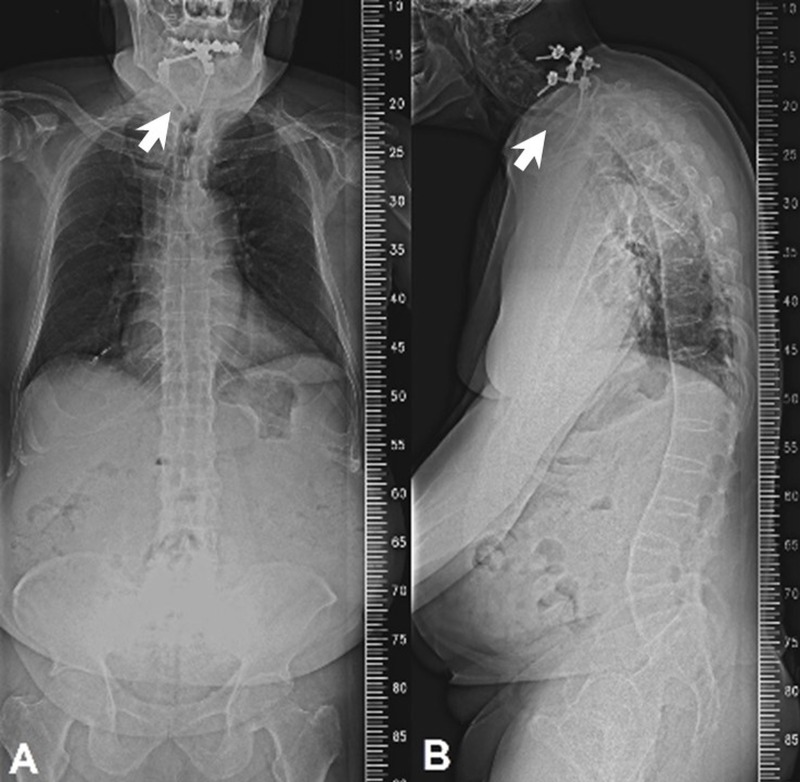
36-inch radiograph down to the pelvis A: Anterior-posterior view of a 36-inch radiograph down to the pelvis, demonstrates no visible cage in the chest or abdomen. B: Lateral view of a 36-inch radiograph down to the pelvis, demonstrates no visible cage in the chest or abdomen.

**Figure 5:**
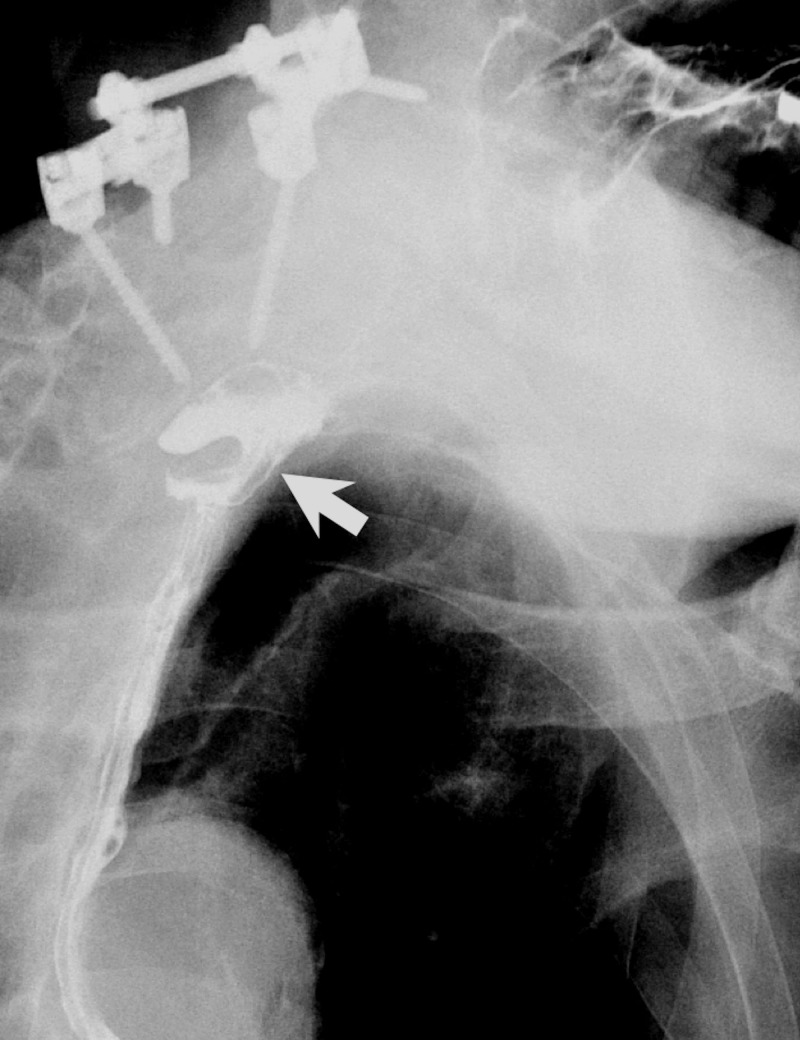
A barium swallowing test of this patient A barium swallowing test demonstrated transient localized contrast accumulation in the oropharynx (arrow).

Surprisingly, the metallic cage disappeared on a subsequent radiograph obtained at 18 months after the second surgery (Figure 3A). The patient had no weakness, nor signs of spinal cord compression. The serial image studies showed no interval changes of posterior screws except the disappeared metallic cage. The MRI scans also confirmed that the cage was no longer visible at the interbody space, nor around the sub-axial pre-vertebral space, or anywhere in the vicinity (Figures 3B-3C). The patient underwent thorough evaluations in an attempt to locate the missing metallic corpectomy cage, including 36-inch radiographs (Figure 4). However, none of the examinations could identify the metallic cage. An upper-gastrointestinal pan-endoscopy was also performed, but neither scars nor perforation signs were identified at the oropharyngeal mucosa. A Barium swallowing test demonstrated a pouch extending from the posterior wall of the cervical esophagus with the transient accumulation of contrast media (Figure 5) which could be thought of as the possible perforation region, although the patient denied any symptoms. Throughout the entire process, since the first surgery, the patient denied any symptoms of gastrointestinal problems or discomfort during defecation. He had never experienced nor noticed a foreign body coming out from the mouth or anus. There was absolutely no other surgery or endoscopic procedures for the removal of the cage during the whole course. Therefore, we reasonably infer that the metallic corpectomy cage may have perforated through the esophagus and had been eliminated through the gastrointestinal tract during defecation without symptoms. To date, the patient has remained free of neurological or gastrointestinal symptoms.

## Discussion

The authors reported on a patient with ankylosing spondylitis who underwent C6 corpectomy and posterior decompression and fixation. The metallic corpectomy dislodged within one month and disappeared within 18 months postoperation. The patient had no dysphagia, did not choke, and had never noticed any objects coming out per os or per anus. Due to the metallic nature and substantial size of the corpectomy cage, it should be easily located by radiological evaluations if it was still inside the body. This is the first report of such a complication - the disappearance of a sizable metallic cage, without symptoms and awareness.

Instrumented anterior cervical fusions, including ACDF and ACCF, have been considered as a standard surgical treatment in the management of a variety of spinal diseases. For example, disc herniation, OPLL, trauma, and spondylosis that cause myelopathy, radiculopathy, or both are common indications for surgery. With instrumentation (i.e., anterior plate and screws), fusion rates reportedly have reached 98% [15-16], with minimized graft-related complications and reduced chances of late deterioration of the cervical spinal alignment [17]. Although very rare, there have been a few reports of esophageal tear or perforation. An iatrogenic esophageal tear might occur during the anterior cervical approach, with particularly higher chances in revision surgery. There have been reports of delayed esophagus or pharyngeal perforation caused by chronic inflammation or infection, which could be associated with subsequent dislodgment and migration of implants and screws [17-18]. On the other hand, the dislodgement of implanted screws, cages, or plates also could cause chronic inflammation and lead to delayed esophageal perforation. These dislodged implants were reportedly located within the gastrointestinal tract, the mediastinum, or even the intraperitoneal space. In 2006, Fountas et al. reported a case of screw loosening and migration into the gastrointestinal tract 13 months after the surgery [4]. In a case reported by Roberto-Gazzeri et al., the screw used with an anterior cervical plate was found as an extrusion 11 years after the surgery, the screw was no longer visible a few days later, and finally was found by radiograph in the right lower abdominal quadrant [5]. Furthermore, Chataigner et al. described a case of a loosened screw that had migrated into the gastrointestinal tract without symptoms three months after surgery [19].

In the present case, the dislodged metallic cage was never located by any of the radiological image studies. The implanted metallic cage of corpectomy was of substantial size, measuring at 11x14x33.5 mm. However, the patient denied any discomfort in his gastrointestinal system nor during defecation since the operations. Such asymptomatic expulsion of the implants used for spinal fusion was indeed very rare. Alfredo-Pompili et al. had reported a case in which an implanted screw was missing, and they assumed that the missing screw caused asymptomatic perforation of the intestinal mucosa and was then eliminated through the gastrointestinal tract [17]. Martinez-Lage et al., Calgi et al., and Guner et al. also relatively reported cases of displaced screws, which were eventually missing in the images during follow-ups in 2007, 2009, and 2014, respectively [1,9]. Furthermore, there have been reports of iatrogenic esophageal perforation after anterior cervical surgery, which were mainly related to infectious processes, including abscess, mediastinitis, and sepsis. However, such perforations might also heal spontaneously with few symptoms. In contrast, delayed esophageal perforation could be problematic. One of the common reasons for delayed esophageal perforation is displaced implants. For example, M Hanci et al. mentioned in 1995 that esophageal perforation might come from pressure caused by metallic implants and micro-trauma effects [20]. Those cases of delayed esophageal perforation that were reported without clinical symptoms nor awareness by the patients were by Yee et al., Chataigner et al., and Guner et al., respectively [19]. In this report, the patient had only complained of slight dysphagia immediately after the first surgery, which was likely attributed to the paralysis of the right-side vocal cord. There was no esophageal scarring identified through the pan-endoscopy of the upper gastrointestinal tract. The migration could have taken place in a very slow fashion so that the patient well-tolerated it.

This is a report of only one case. Although the patient was free of neurological or gastrointestinal symptoms, the consequences could have been much worse. The lesson was that any failure to achieve arthrodesis in ACDF or ACCF should be monitored if not revised by a secondary operation.

## Conclusions

The complete dislodgement of a cervical corpectomy cage that was placed anteriorly could occur without symptoms. Fortunately, the sizable metallic cage might have been expelled during bowel movement and caused little problems. Therefore, failure to achieve arthrodesis in anterior cervical fusion must be closely monitored.

## References

[1] Cagli S, Isik HS, Zileli M (2009). Cervical screw missing secondary to delayed esophageal fistula: case report [Article in English, Turkish]. Turk Neurosurg.

[2] Schulz C, Mauer UM, Mathieu R (2017). Clinical and radiological results after anterior cervical corpectomy with cage fusion - a retrospective comparison of PEEK vs titanium cages [Article in English, German]. Z Orthop Unfall.

[3] Buttermann GR (2017). Anterior cervical discectomy and fusion outcomes over 10 years: a prospective study. Spine (Phila Pa 1976).

[4] Fountas KN, Kapsalaki EZ, Machinis T, Robinson JS (2006). Extrusion of a screw into the gastrointestinal tract after anterior cervical spine plating. J Spinal Disord Tech.

[5] Gazzeri R, Tamorri M, Faiola A, Gazzeri G (2008). Delayed migration of a screw into the gastrointestinal tract after anterior cervical spine plating. Spine (Phila Pa 1976).

[6] Geyer TE, Foy MA (2001). Oral extrusion of a screw after anterior cervical spine plating. Spine (Phila Pa 1976).

[7] Leven D, Cho SK (2016). Pseudarthrosis of the cervical spine: risk factors, diagnosis and management. Asian Spine J.

[8] Piazza BR, Pace GI, Knaub MA, Bible JE (2017). Anterior cervical discectomy and fusion pseudarthrosis: posterior versus "redo" anterior. Clin Spine Surg.

[9] Martinez-Lage JF, Felipe-Murcia M, Martinez-Lage Azorin L (2007). Late prevertebral abscess following anterior cervical plating: the missing screw. Neurocirugia (Astur).

[10] Koehler S, Raslan F, Stetter C, Rueckriegel SM, Ernestus RI, Westermaier T (2015). Autologous bone graft versus PEKK cage for vertebral replacement after 1- or 2-level anterior median corpectomy. J Neurosurg Spine.

[11] Kotil K, Tari R (2011). Two level cervical corpectomy with iliac crest fusion and rigid plate fixation: a retrospective study with a three-year follow-up. Turk Neurosurg.

[12] Schulz C, Mauer UM, Mathieu R (2017). PEEK cage fusion after anterior cervical corpectomy: clinical and radiological results in patients with spondylotic myelopathy [Article in English, German]. Der Orthopade.

[13] Lippman CR, Hajjar M, Abshire B, Martin G, Engelman RW, Cahill DW (2004). Cervical spine fusion with bioabsorbable cages. Neurosurg Focus.

[14] Pflugmacher R, Schleicher P, Gumnior S (2004). Biomechanical comparison of bioabsorbable cervical spine interbody fusion cages. Spine (Phila Pa 1976).

[15] Bose B (1998). Anterior cervical fusion using Caspar plating: analysis of results and review of the literature. Surg Neurol.

[16] Caspar W, Barbier DD, Klara PM (1989). Anterior cervical fusion and Caspar plate stabilization for cervical trauma. Neurosurgery.

[17] Pompili A, Canitano S, Caroli F, Caterino M, Crecco M, Raus L, Occhipinti E (2002). Asymptomatic esophageal perforation caused by late screw migration after anterior cervical plating: report of a case and review of relevant literature. Spine (Phila Pa 1976).

[18] Fuji T, Kuratsu S, Shirasaki N (1991). Esophagocutaneous fistula after anterior cervical spine surgery and successful treatment using a sternocleidomastoid muscle flap. A case report. Clin Orthop Relat Res.

[19] Chataigner H, Gangloff S, Onimus M (1997). Spontaneous elimination by the natural tracts of screws of anterior cervical osteosynthesis. Apropos of a case [Article in French]. Rev Chir Orthop Reparatrice Appar Mot.

[20] Hanci M, Toprak M, Sarioglu AC, Kaynar MY, Uzan M, Islak C (1995). Oesophageal perforation subsequent to anterior cervical spine screw/plate fixation. Paraplegia.

